# Ability to Maintain Internal Arousal and Motivation Modulates Brain Responses to Emotions

**DOI:** 10.1371/journal.pone.0112999

**Published:** 2014-12-01

**Authors:** Virginie Sterpenich, Sophie Schwartz, Pierre Maquet, Martin Desseilles

**Affiliations:** 1 Cyclotron Research Centre, University of Liege B30, Liege, Belgium; 2 Department of Neuroscience, University of Geneva, Geneva, Switzerland; 3 Swiss Center for Affective Sciences, University of Geneva, Geneva, Switzerland; 4 Department of Neurology, CHU Sart-Tilman, University of Liege, Liege, Belgium; The University of Kansas Medical Center, United States of America

## Abstract

Persistence (PS) is defined as the ability to generate and maintain arousal and motivation internally in the absence of immediate external reward. Low PS individuals tend to become discouraged when expectations are not rapidly fulfilled. The goal of this study was to investigate whether individual differences in PS influence the recruitment of brain regions involved in emotional processing and regulation. In a functional MRI study, 35 subjects judged the emotional intensity of displayed pictures. When processing *negative* pictures, low PS (vs. high PS) subjects showed higher amygdala and right orbito-frontal cortex (OFC) activity but lower left OFC activity. This dissociation in OFC activity suggests greater prefrontal cortical asymmetry for approach/avoidance motivation, suggesting an avoidance response to aversive stimuli in low PS. For *positive* or *neutral* stimuli, low PS subjects showed lower activity in the amygdala, striatum, and hippocampus. These results suggest that low PS may involve an imbalance in processing distinct emotional inputs, with greater reactivity to aversive information in regions involved in avoidance behaviour (amygdala, OFC) and dampened response to positive and neutral stimuli across circuits subserving motivated behaviour (striatum, hippocampus, amygdala). Low PS affective style was associated with depression vulnerability. These findings in non-depressed subjects point to a neural mechanism whereby some individuals are more likely to show systematic negative emotional biases, as frequently observed in depression. The assessment of these individual differences, including those that may cause vulnerability to depressive disorders, would therefore constitute a promising approach to risk assessment for depression.

## Introduction

Although population studies have repeatedly demonstrated that emotion processing and regulation involve specific brain regions [1], emotional responses also vary considerably across individuals [2]. At the behavioural level, the quality and intensity of emotional and affective reactions to comparable stimuli vary highly across individuals, and have been referred to as “affective styles” or temperaments [3]. Affective styles have been distinguished in terms such as inhibited vs. uninhibited temperament [4], extraverted vs. introverted personality [5], and vulnerability to psychopathology [6]. These differences are not unique to humans, but are also found in several animal species [7]. Recently, biological temperament substrates have been documented and related to neurotransmission mechanisms [8], genetic characteristics [9], electroencephalography [10], and brain imaging correlates (e.g., [11]).

Clinical and experimental studies indicate that persistence (PS), a subscale of the Temperament and Character Inventory – Revised (TCI–R) scale developed by Cloninger [11], is an independent personality dimension. Cloninger defines PS as the ability to generate and maintain arousal and motivation internally in the absence of immediate external reward [12]. High PS individuals are generally diligent, hardworking, industrious, ambitious, overachieving, and perfectionist [12]. In contrast, low PS individuals tend to get discouraged and give up when expectations are not fulfilled rapidly, rather than persevering despite frustration [13], [14]. Low PS individuals cannot maintain arousal and motivation in the absence of external incentives, and could therefore be more dependent on positive stimuli and more vulnerable to negative stimuli.

In TCI–R-based studies, low PS was consistently found in subjects with major depressive disorders [15], [16] and bipolar disorders (type I or II) [16] compared to controls. Outside the field of mood disorders, PS has also been shown to be involved in other pathophysiologies, such as attention deficit hyperactivity disorder (ADHD) [17].

Cloninger proposes that PS can be measured by the partial reinforcement extinction effect (PREE), whereby persistent subjects are more resistant to extinction of previously intermittently rewarded behaviours than controls who have been continuously reinforced [13], [18]. Moreover, a PREE-based treatment strategy designed to foster persistence has been proposed, called “persistence training” [19], [20]. In rodents, the PREE is dependent on the integrity of the nucleus–hippocampus pathway [21]. Nucleus accumbens activity has been correlated with PS scores in humans [11]. In addition, Cloninger proposed that the connection between the hippocampus, also involved in memory and emotions in interaction with the amygdala [22], and the nucleus accumbens might allow a conditioned signal of punishment to change into a conditioned signal of anticipated reward [13]. Osher et al. [18] suggested that euthymic bipolar patients, who were found to be low in PS compared to controls, are unusually susceptible to switches between behavioural activation and inhibition under intermittent positive reinforcement

Over the last decade, functional magnetic resonance imaging (fMRI) studies have substantially refined our knowledge of the networks involved in emotion recognition [23] and attention [24]. These networks include the amygdala, ventral striatum, and orbitofrontal cortex, among others. For example, using fMRI, Gusnard et al. [11] found that individual differences in PS were associated with differential brain activation in emotion regulation networks (including the lateral orbital and medial prefrontal cortex [mPFC] and ventral striatum) in a self-referential judgment task (how the picture made them feel) [11]. A correlational analysis showed higher activity in the orbital and adjacent mPFC and ventral striatum in highly PS subjects, with lower activity in low PS subjects. In addition, the amygdala, anterior cingulate cortex (ACC), and ventral striatum were co-activated with the anterior insular cortex in most imaging studies of emotion (e.g., [25]). The close interactions between the emotional salience that is represented by amygdala activity, the volitional urges represented by ACC activity, the incentive signals represented by ventral striatum activity, and the feelings state represented by anterior insular cortex activity suggest that each global emotional moment integrates feelings and motivational values [25]. The PFC was shown to be involved in an approach/avoidance system whereby left frontal regions contribute to approaching positive stimuli and right frontal regions to avoiding negative stimuli [26]–[28]. The present study focuses on a network of brain areas involved in emotion regulation and interoception. These areas specialize in distinguishing positive and negative rewards, are involved in approach- and avoidance-related affect, and are crucial for feelings and motivations related to bodily needs.

The goal of this fMRI study was not to experimentally manipulate motivation processes per se, but to investigate how individual differences in PS are related to the automatic engagement of distinct emotion regulation mechanisms to process emotional information [29]. As a critical control for the selectivity of our results with respect to PS, the two groups of subjects differed on the PS score only, and not on depression, anxiety, or alexithymia scores. Subjects were scanned while they judged the emotional valence of pictures, without actively engaging in a reappraisal strategy. Changes in pupillary diameter measure the autonomic response, including modulations of emotional arousal. This physiological response changes rapidly, and can be used to assess emotional arousal elicited by each distinct stimulus. A study using pupillary diameter found a correlation between responses in the anterior cingulate cortex and autonomic arousal during a Stroop inhibitory task (Critchley et al., 2005). We used this measure to assess trial-wise changes in emotional arousal. Using fMRI, we measured changes in both local brain activity and functional connectivity between regions. Three main improvements were introduced in this study over previous studies. First, we used an event-related fMRI design (as opposed to a block design [11]) with emotional (and non-emotional) pictures presented in random order to prevent the use of voluntary cognitive strategies (e.g., suppressing emotional reactions to aversive stimuli) and to minimize emotional adaptation effects (e.g., in the amygdala [30]). Second, unlike Gusnard et al. who examined evoked BOLD response to emotional stimuli as a function of the percentage of trials in a given block that were neutral, in our experiment stimuli of different emotions were mixed according to an event-related design, and the impact of persistence was assessed for stimuli of each emotional condition (negative, positive, and neutral) separately [11]. Finally, whereas biological emotion regulation systems have been proposed in the literature [31], [32], it remains unclear whether these systems also come into play when emotional information is processed in the absence of cognitive reappraisal instruction.

Our hypothesis is that low PS subjects viewing emotional or neutral pictures in the absence of instructions to increase or decrease their emotional valence would show a spontaneous increase in activity in brain areas involved in emotion perception (amygdala) because they are more vulnerable to negative stimuli, and a decrease in regions involved in emotion regulation for negative items (OFC) because they tend to become discouraged. Low PS subjects should also show lower activity in regions involved in memory (hippocampus) and reward processing (amygdala or striatum) when processing neutral or positive information because they have difficulty maintaining arousal and motivation.

## Material and Methods

### 2.1 Subjects

This study employs a data set for which the group-average data have been reported previously [33]. Subjects provided their written informed consent to participate in this functional magnetic resonance imaging (fMRI) study, which was approved by the Ethics Committee of the Faculty of Medicine of the University of Liège. All subjects completed the Beck Depression Inventory [34], the Beck Anxiety Inventory, and the Bermond-Vorst Alexithymia Questionnaire [35]. Between-group comparisons were conducted using Tukey's Honestly Significant Difference (HSD) test.

#### Persistence and the Temperament and Character Inventory – Revised (TCI–R)

Persistence was derived from the responses to the 35 questions contained in the TCI–R. This self-report inventory based on a seven-factor model was used to characterize subjects' personality. They responded to the 35 items of the French version of the TCI–R at home. The TCI–R was translated from the original English version by Pélissolo, Notides, Musa, Téhérani, and Lépine (2000) [36]. We chose to use the TCI–R over the TCI because the revised scale has similar psychometric characteristics to those of the initial version, but with a significantly improved factorial structure and internal consistency, particularly for PS. Perlissolo et al. proposed that the psychometric properties and predictive values of the TCI–R for the persistence scale were superior to those for the former version [37], and to the Tridimensional Personality Questionnaire, the initial version of the TCI.

### 2.2 Stimuli and behavioural task

The stimulus set was taken from the International Affective Pictorial System [38]. It consisted of 160 emotional pictures (80 unpleasant, mean valence on a 9-point scale: 2.87±0.66; 80 pleasant, mean valence: 7.4±0.48) and 80 neutral pictures (mean valence: 5.4±0.65). Each valence category contained a similar proportion of objects, landscapes, animals, and human beings. Picture luminance was equalized to obtain the same mean luminance for all pictures.

In the fMRI session, 40 pictures representing each valence (unpleasant, pleasant, neutral) were randomly selected for each subject and presented in random order. Each picture was displayed for 3 s (17°×23° of visual angle). After the stimulus disappeared, the subjects had a maximum of 8 s to rate the emotional valence on a 7-point scale (“–3”: very unpleasant, “0”: neutral, “+3”: very pleasant) by pressing on two hand-held keypads. Between trials, a fixation cross (3.75°×3.75° of visual angle) was displayed on a light grey background for 1.5 seconds to ensure pupillo-constriction, allowing better detection of pupillary dilatation in relation to the stimulus presentation. Forty null events, consisting of presentation of the fixation cross for 6 s, were randomly introduced between trials [39].

### 2.3 Functional MRI data acquisition

Data were acquired with a 3T head-only magnetic resonance (MR) scanner (Allegra; Siemens, Erlangen, Germany) using a gradient echo-planar imaging (EPI) sequence (32 transverse slices with 30% gap; voxel size, 3.4×3.4×3.4 mm; repetition time (TR), 2130 ms; echo time (TE), 40 ms; flip angle, 90°; 220 mm field of view (FOV)). From 420 to 480 functional volumes were acquired over the session, with the first three volumes discarded to account for magnetic saturation effects. A structural MR scan was acquired at the end of the experimental session (T1-weighted 3D MP-RAGE sequence, TR: 1960 ms, TE: 4.43 ms, TI: 1100 ms, FOV: 230×173 mm^2^, matrix size 256×192×176, voxel size: 0.9×0.9×0.9 mm). Stimuli were displayed on a screen positioned at the rear of the scanner, which the subject could comfortably see by means of a mirror mounted on the standard head coil.

### 2.4 Functional MRI data analysis

Functional MRI data were analysed using SPM2 (http://www.fil.ion.ucl.ac.uk) in MATLAB (Mathworks Inc., Natick, MA). Functional scans were realigned using iterative rigid body transformations that minimize the residual sum of squares between the first and subsequent images. They were then normalized to the MNI EPI template and resampled to a voxel size of 2×2×2 mm (2D spline) and spatially smoothed with a Gaussian kernel with full-width at half maximum (FWHM) of 8 mm.

Data were processed using a two-step analysis, taking into account intra-individual and inter-individual variance, respectively. For each subject, brain responses were modelled at each voxel using a general linear model. Three trial types were modelled: negative (neg), positive (Pos), and neutral (Neu) images. Each trial was categorized as neutral or emotional based on individual subjective ratings. Negative images corresponded to the responses “−3” and “−2”, neutral images to “−1”, “0”, and “+1”, and positive images to “+2” and “+3”. For each trial type, the onsets for each picture were modelled as a delta function and used as a regressor in the individual design matrix. Movement parameters estimated during realignment (translations in x, y, and z directions and rotations around x, y, and z axes) and a constant vector were also included in the matrix as a variable of no interest. High pass filter was implemented using a cut-off period of 128 s to remove low frequency drifts from the time series. Serial autocorrelations were estimated with a restricted maximum likelihood algorithm using an autoregressive model of order 1 (+ white noise). Linear contrasts estimated the main effects of emotion: (negative vs. neutral), (positive vs. negative), (positive vs. neutral), (neutral vs. [negative + positive]), and common effect of negative, positive, and neutral). The resulting voxel set constituted maps of t statistics [SPM(T)]. Individual summary statistical images were spatially smoothed with a Gaussian kernel with FWHM of 6 mm. Multifiltering allows greater sensitivity when various signal types are present in brain activation images (for a similar approach, see [40]). Contrast images were used in a second-level (random-effect) analysis. Note that even if the affective judgment task did not include a baseline condition, the baseline was modelled implicitly (i.e., the implicit baseline is whatever is not included in the model) and robustly in the design matrix by using lengthy fixation periods between events (40 null events, corresponding to a fixation cross of 6 s, were introduced randomly between trials) [41].

The second-level analysis consisted first in one-sample t-tests for the main effect of emotions across all subjects. We then used two-sample t-tests to compare responses between the two subject groups according to PS score (low PS> high PS and low PS <high PS). To exclude from the main analysis voxels that show significant effects of anxiety, alexithymia, or depression, we performed an exclusive masking procedure. Thus, in addition to persistence, we computed similar analyses for three other questionnaires (anxiety, alexithymia, and depression), splitting the population into two groups around the median for each questionnaire. Two-sample t-tests were then performed to compare the two groups on the contrasts of interest. For example, we exclusively masked the contrast [neg> neu, low PS> high PS] by the other contrasts: [neg> neu, high> low anxiety], [neg> neu, high> low alexithymia] and [neg> neu, high> low depression], meaning that regions more activated in low than high PS subjects for negative pictures are not activated in the contrast neg> neu in high vs. low anxiety subjects, or high vs low depression or high vs low alexithymia. The masks were threshold at a lenient threshold (p<0.05 uncorrected). We performed a whole-brain analysis followed by small volume correction for a priori regions that were previously shown to be activated by emotional stimuli. The contrasts of interest corresponded to an emotion x PS status interaction. The resulting set of voxel values was thresholded at p<0.001 (uncorrected) and with a minimum cluster size of 5 voxels. Statistical inferences were corrected for multiple comparisons using Gaussian random field theory at the voxel level in a small spherical volume (radius 10 mm) around a priori locations of structures of interest, taken from the literature (and specified in the tables). This published information collected in independent samples on similar tasks constitutes prior spatial information that usefully constrains statistical inferences. A priori brain regions are those involved in the perception of negative emotion (amygdala, orbitofrontal cortex, insula [23], and locus coeruleus [42]) in the perception of positive emotion (ventral striatum and caudate nucleus) and emotion-modulated regions involved in vision (occipital cortex) [43] and memory (hippocampus) [43].

### 2.5 Psychophysiological interaction analyses

Psychophysiological interaction (PPI) analyses were computed to test the hypothesis that functional connectivity between seed regions observed in the contrasts of interest (orbitofrontal and right amygdala for Neg> Neu; hippocampus and left amygdala for Pos> Neg, see results) and the rest of the brain as a function of emotional conditions was also influenced by subjects' PS (low PS vs. high PS, high vs. low PS, respectively). For each individual, the coordinates of the seed area corresponded to the local maxima (single voxel) detected within a 10 mm radius sphere of the peak voxel of the group analysis. A new linear model was prepared for PPI analyses at the individual level, using three regressors. One regressor represented the emotional status of pictures (neg vs. neu or pos vs. neu), and the second regressor was the raw time series of activity in the reference areas during testing. The third regressor represented the interaction of interest between the first (psychological) and the second (physiological) regressors. To build this regressor, the underlying neuronal activity was first estimated by a parametric empirical Bayes formulation combined with the psychological factor and subsequently convolved with the hemodynamic response function [44]. The model also included movement parameters. A significant psychophysiological interaction indicated a change in the regression coefficients between any reported brain area and the reference region related to the presentation of negative vs. neutral (or positive vs. neutral) stimuli. Next, individual summary statistic images obtained at the first-level (fixed-effects) analysis were spatially smoothed (6 mm FWHM Gaussian kernel) and entered in a second-level (random-effects) analysis using two sample t-tests to compare functional connectivity between groups (low> high and low <high). Inferences were conducted as for the main effect analysis.

### 2.6 Analysis of behavioural data

Items were split according to the subjective emotion rating by each subject (negative  =  responses “−3” or “−2”; neutral  =  responses “−1”, “0”, or “+1”; positive  =  responses “+2” or “+3”). Two repeated measure ANOVAs with emotion (neg, neu, pos) as within-subject factors and PS group (low vs. high) as the between-subject factor were performed to test the effects of PS, emotion, and their interaction. The first ANOVA used the number of images identified for each emotion, and the second analysis used the reaction time for each emotion type.

### 2.7 Acquisition and analysis of pupillary size data

During data acquisition, eye movements and pupillary size were measured continuously with an infrared eye tracking system (LRO5000, ASL, Bedford, MA, sampling rate: 60 Hertz). Mean pupillary size was estimated over one second immediately following picture display onset. During this interval, the pupillary size was sufficiently stable to assess the autonomic arousal, which was expected to be modulated primarily by the emotional intensity of the images. Trials contaminated by blinks were discarded. To reduce inter-subject variability, baseline pupillary size was estimated during null events (fixation cross) for each subject, averaged, and subtracted from mean individual values. A repeated measure ANOVA with emotion (Neg, Neu, Pos) as within-subject factors and PS (low vs. high) as the between-subject factor tested for the effects of emotion, PS, and their interaction. Separate trials were also run for the three emotions according to individual subjective ratings. Planned comparisons tested the differences between negative vs. neutral and positive vs. neutral pupillary size.

## Results

### 3.1 Population

Thirty five right-handed normal-sighted healthy subjects (20 females; mean age, 22.3±2.8 years) participated in this study. No subjects had medical, psychiatric, or traumatic history. Scores for the whole population on the Beck Depression Inventory (4.0±4.1) [34], the Beck Anxiety Inventory (6.2±5.8), and the Bermond-Vorst Alexithymia Questionnaire (total score: 51.7±16.5) [37] were within normal range. Subjects were divided into two groups based on a median split on the persistence score: 17 low PS, subjects(10 females, 7 males; mean age: 21.1±2.6; PS scores: 85–118; mean PS: 111.4±8.3), and 17 high PS subjects (9 females, 8 males; mean age 23.2±2.8; PS score: 120–150; mean PS: 130.2±8.8). One participant with a score equal to the median was excluded from further analysis, leaving a final sample of 33 subjects. The two groups differed on the PS score, but not on depression (p = 0.43), anxiety (p = 0.09), or alexithimia scores (p = 0.28).

### 3.2 Behavioural data

During the fMRI session, subjects had to rate the emotional valence of pictures on a 7-point scale ranging from −3 to +3 (Figure S1). An ANOVA performed on the number of images identified as negative (−3, −2), neutral (−1, 0, +1), or positive (+2, +3) as within-subject factors and PS as the between-subject factor revealed no significant between-group difference (F(1,32)  = 0.22, p = 0.64, Table S1 in File S1), a significant difference between emotions (F(2,64)  = 49.97, p<0.001) because participants rated more images as neutral than negative or positive (note that this effect reflects our grouping of the responses into three emotional categories, with more neutral responses), and no group by emotion interaction (F(2,64)  = 1.09, p = 0.34). A second ANOVA with the mean reaction time for the three emotion categories as within-subject factors and PS group as the between-subject factor showed an effect of emotion (F(2,64)  = 11.51, p<0.001) because participants took more time to report the emotion for neutral pictures, no group effect (F(1,32)  = 1.29, p = 0.29), and no interaction between emotion and the PS group (F(2,64)  = 0.80, p = 0.45). Taken together, these behavioural results suggest that PS does not significantly affect the explicit judgment of emotional pictures. In the fMRI analysis, we assigned each picture (i.e., each trial) to one of three emotional categories (Neg, Neu, Pos) based on subjects' individual judgments during the task. Critically, mean IAPS valence and arousal ratings for the three categories based on subjects' self-ratings did not differ between high and low PS subjects.

### 3.3 Pupillary size data

Pupillary size (Table S1 in File S1) can reflect autonomic reactivity, and can therefore be used as an objective physiological measure of emotional arousal. We performed an ANOVA with mean pupillary size measured for each emotion during picture presentation as the within-subject factors and PS group as the between-subject factor. We observed a main effect of emotion (F(2,64)  = 58.44, p<0.001), but no effect of PS group (F(1,32)  =  0.67, p = 0.42) and no emotion by PS interaction (F(2,64)  = 1.82, p = 0.17). Post-hoc planned comparison analyses revealed that negative pictures induced larger pupillary size compared to neutral pictures (F(1,32)  = 106.1, p<0.001) and positive pictures (F(1,32)  = 92.26, p<0.001) in both groups, and that positive pictures were not more strongly associated with larger pupillary size compared to neutral pictures (F(1,32)  = 0.58, p = 0.45) for both groups. More importantly, differences in pupillary size between negative and positive pictures tended to be larger in the low PS group than in the high PS group (F(1,32)  = 3.31, p = 0.08).

### 3.4 Functional MRI data

Because they are not the main focus of this study, we report the main effects of emotion for the whole population (negative vs. neutral (Table S3 in File S1), negative vs. positive (Table S4 in File S1), positive vs. neutral (Table S5 in File S1), and positive vs. negative (Table S6 in File S1)). The results of interest concern the interaction between the main effects of emotion and individual PS scores, as described in detail below.

Importantly, we used exclusive masks to exclude areas involved in anxiety, alexithymia, and depression. This procedure ensures that, when comparing high and low PS subjects, we selectively report regional changes in brain activity due to PS only, and not attributable to the other psychological dimensions addressed.

#### 3.4.1 Activity and functional connectivity changes for negative (vs. neutral) stimuli

**Figure 1:**
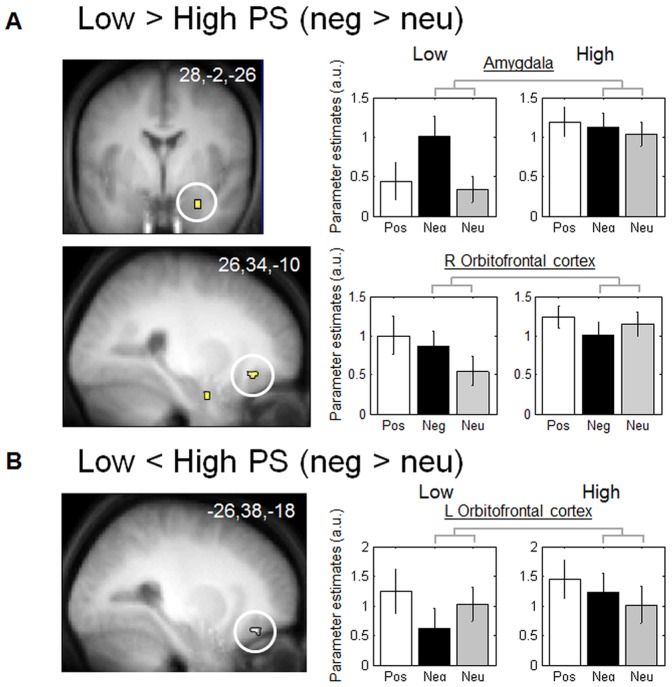
Functional MRI responses to negative vs. neutral pictures. **A**: Brain regions significantly more activated in low PS subjects compared to high PS subjects. The right amygdala and right orbitofrontal gyrus and their parameter estimates. **B.** Brain regions significantly deactivated in low PS subjects compared to high PS subjects. The left orbitofrontal gyrus and its parameter estimates.

Using two-sample t-tests, we first assessed how regional brain responses to negative pictures (Neg – Neu) differed between low and high PS groups. Compared to high PS, low PS subjects showed strong differential activation in the amygdala for negative compared to neutral pictures (Table 1 and Figure 1A), in contrast to high PS subjects (Table 1 and Figure 1B). A similar effect was observed in the right orbitofrontal cortex (Figure 1A) and middle occipital cortex (Table 1). Low PS subjects showed less activation in the left orbitofrontal cortex for negative pictures (vs. neutral pictures) (Table 1 and Figure 1B) compared to high PS subjects. The parameter estimates demonstrated that this region was less activated for negative than neutral stimuli in low PS subjects, with the inverse effect in high PS subjects.

**Table 1:** Functional MRI responses to negative vs. neutral pictures.

Name of brain region	MNI coordinates (x,y,z, mm)	Side	Z-score	Cluster size	Psvc	Coordinates from the literature
**Low> High PS**						
Right Orbitofrontal cortex	26,34,−10	R	3.56	8	0.015	[72] 30, 40, −16
Amygdala	28,−2,−26	R	3.17	7	0.044	[73] 34,−4, −30
Middle occipital gyrus/angular gyrus	34,−76,34	R	3.17	68	0.044	[74] −40,−70,30
**Low <High PS**						
Left Orbitofrontal cortex	−26,38,−18	L	3.36	7	0.026	[72] −30,40,−16

We then tested whether the regions of interest that were significantly more or less activated in low PS subjects, according to the above comparisons, would establish distinct functional connections with other brain regions when processing negative vs. neutral pictures and according to low or high PS. The seed regions were the right amygdala identified in the contrast (Neg> Neu) x (Low> High) and the left orbitofrontal cortex identified in the contrast (Neg> Neu) x (Low <High). The right amygdala was found to be more closely connected to the anterior insula and the fusiform (Table S2 in File S1 and Figure S2A) in low compared to high PS subjects, with a stronger connection for negative than neutral images. On the other hand, the left orbitofrontal cortex was more closely connected to several occipital regions (superior occipital gyrus, superior lingual gyrus, and cuneus) (Table S2 in File S1, Figure S2B) in high compared to low PS subjects, with stronger connections for negative than neutral images. As shown by the parameter estimates, these regions were more connected to the orbitofrontal cortex in the high PS compared to the low PS group.

#### 3.4.2 Activity changes for positive (vs. negative) stimuli

**Figure 2:**
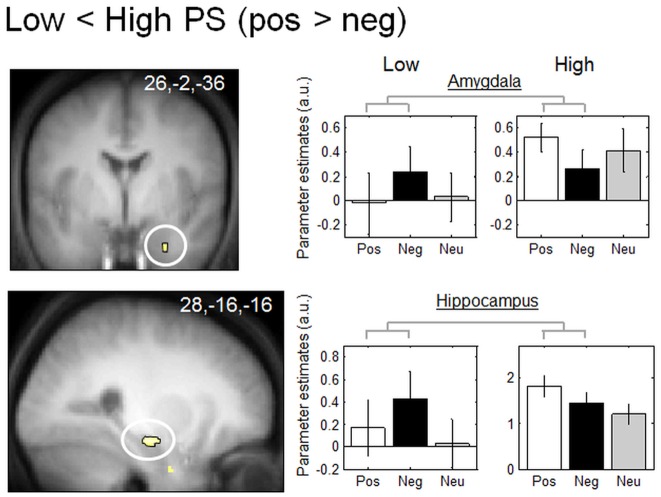
Functional MRI responses to positive vs. negative pictures. Brain regions significantly deactivated in low PS subjects compared to high PS subjects: the right amygdala and right hippocampus. Functional results are displayed on the mean structural MR image of subjects normalized to the MNI stereotactic space (display at p, 0.001, uncorrected). Parameter estimates are calculated for positive (Pos) and negative (Neg) items. Arbitrary units, error bars: SEM.

To isolate a specific effect of emotional valence, we tested for regions that were differentially activated during the presentation of positive (compared to negative) pictures in low and high PS subjects. When viewing positive (compared to negative) pictures, low PS subjects showed significantly lower activation of the right amygdala and right hippocampus compared to high PS subjects (Table 2 and Figure 2). The parameter estimates indicate that these two brain regions were more deactivated for positive than negative pictures in low PS subjects only, with the reverse pattern for high PS subjects. However, the contrast low> high in PS subjects for positive vs. negative pictures showed no significant brain activation.

**Table 2:** Functional MRI responses to positive vs. negative pictures.

Name of brain region	MNI coordinates (x,y,z, mm)	Side	Z-score	Cluster size	Psvc	Coordinates from the literature
**Low> High PS: No significant activation**						
**Low <High PS**						
Hippocampus	28,−16,−16	R	3.44	32	0.019	[75] 22, −16, −12
Amygdala	26,−2,−36	R	3.43	5	0.044	[73] 22, 0, −30

The contrast positive vs. neutral stimuli showed increased but not statistically significant activity in regions similar to those found in the contrast positive vs. negative stimuli. For example, the hippocampus (32, −14, −14, Z-score  = 2.43, p = 0.008 uncorrected) and left amygdala (−22,−6,−38, Z-score  = 2.60, p = 0.005 uncorrected) were activated in the contrast positive vs. neutral stimuli. Whereas this confirms a robust network of regions activated by positive stimuli, the contrast positive vs. negative stimuli has the advantage of comparing two emotional stimuli, and therefore represents a positive emotional valence rather than an emotional effect. Note that the contrast Neg> Pos x low> high is equivalent to the contrast Pos> Neg x low <high (Table 2).

PPI analyses performed on the left amygdala (see Material and Methods) showed a significantly larger increase in functional connectivity with the right orbitofrontal cortex (32,54,−6, Z-score  = 3.34, p<0.001 uncorrected) for positive compared to neutral items, and larger for high vs. low PS subjects.

#### 3.4.3 Distinct brain response to neutral (vs. emotional) stimuli

**Figure 3:**
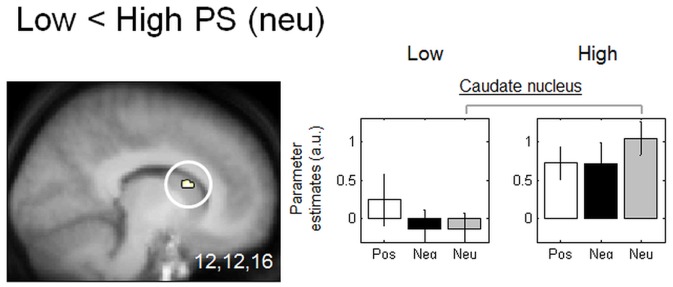
Functional MRI responses to neutral pictures (exclusively masked by negative and positive pictures). Brain regions significantly less activated in low PS subjects compared to high PS subjects: the caudate nucleus. Functional results are displayed on the mean structural MR image of subjects normalized to the MNI stereotactic space (display at p, 0.001, uncorrected). Parameter estimates are calculated for neutral (Neu) items for both subject groups. Arbitrary units, error bars: SEM.

We examined whether low and high PS groups would differ in their brain responses to non-emotional, neutral stimuli. We assessed group differences in the neutral condition while removing any non-specific differences due to a potential baseline shift. We therefore masked (exclusive masking) the contrast high vs. low PS subjects for neutral pictures by combining the contrasts negative vs. baseline (Low <High PS) and positive vs. baseline (Low <High PS). This combination reveals non-specific group effects that are attributable to baseline changes. Masking the contrast for neutral stimuli using this combination therefore removes potential baseline group effects. We found that the caudate nucleus was selectively more activated in the neutral condition in high vs. low PS subjects (Table 3 and Figure 3). The parameter estimates showed that this brain region was less activated in low compared to high PS subjects when neutral images were presented. No region was more activated in low compared to high PS subjects for neutral pictures.

**Table 3:** Functional MRI responses to neutral pictures (exclusively masked by negative and positive pictures).

Name of brain region	MNI coordinates (x,y,z, mm)	Side	Z-score	Cluster size	Psvc	Coordinates from the literature
**Low> High PS: No significant activation**						
**Low <High PS**						
Caudate nucleus	12,12,16	R	3.50	10	0.017	[76] −16, 10, 12

#### 3.4.4 Distinct between-group brain responses, irrespective of stimulus emotion

Finally, we found a main group effect (high vs. low PS), irrespective of displayed picture (neutral, positive, or negative). Specifically, we found increased response to all types of stimuli, i.e. irrespective of the emotional valence (positive, negative, and neutral trials taken together) in the high PS group compared to the low PS group in a set of brain areas involving the insula and amygdala. The locus coeruleus did not survive to our size limit of 5 voxels but was also significant after SVC correction (Table 4 and figure 4). The parameter estimates demonstrated that these regions were more activated in high compared to low PS subjects for the three emotions.

**Table 4:** Functional MRI responses to neutral, positive, and negative pictures.

Name of brain region	MNI coordinates (x,y,z, mm)	Side	Z-score	Cluster size	Psvc	Coordinates from the literature
**Low> High PS: No significant activation**						
**Low <High PS**						
Amygdala	20,−14,−18	R	3.43	21	0.025	[77] −22, −18, −22
Locus coeruleus	−6,−38,−30	L	3.21	4	0.043	[47] 6, −36, −28
Insula	36,0,20	R	4.29	86	0.005	[78] 41,−3,18

**Figure:**
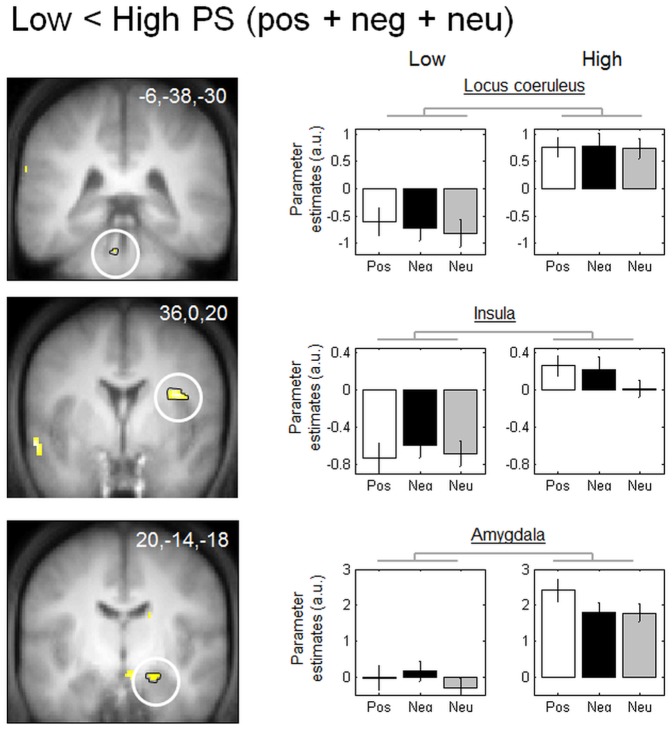
Functional MRI responses to low vs. high PS (regardless of picture valence). Brain regions significantly less activated in low PS subjects compared to high PS subjects: The locus coeruleus, insula, and amygdala. Functional results are displayed on the mean structural MR image of subjects normalized to the MNI stereotactic space (display at p, 0.001, uncorrected). Parameter estimates are calculated for neutral (Neu) items for both subject groups. Arbitrary units, error bars: SEM.

## Discussion

We showed that individual differences in persistence (PS), an independent temperament in Cloninger's TCI–R [14], defined as the ability to generate and maintain arousal and motivation internally in the absence of immediate external reward, influenced the recruitment of brain regions involved in emotion processing and regulation as well as functional interactions between these regions. Importantly, our goal was not to experimentally manipulate motivation processes per se, but to assess the impact of intrinsic motivation as an individual characteristic on emotional processing, similar to studies of individual anxiety and depression [45], [46]. Specifically, we found that low PS subjects showed higher brain response to negative pictures in the amygdala and right OFC, with decreased activity in the left OFC, suggesting increased affective processing and avoidance of aversive stimuli [26]–[28]. Moreover, low PS subjects showed reduced activity in several regions involved in emotional relevance and learning (amygdala, striatum, and hippocampus), specifically when when non-aversive pictures, including positive and neutral pictures, were displayed.

Persistence, as defined by Cloninger, is not equivalent to emotional arousal or autonomous, transient responses to emotions (as indexed by pupillary responses), but it does reflect a general ability to maintain arousal or motivation. We previously showed that pupil diameter increases when subjects are exposed to emotional stimuli, and that this increase correlates with transient activity in the locus coeruleus [47]. Of note, in both groups negative pictures elicited more arousal (i.e., increased pupil diameter) than positive pictures (p<0.001) or neutral pictures (p<0.001), and this difference tended to be larger for low PS vs. high PS, though this difference was not statistically significance (p = 0.088), suggesting that low PS individuals show more contrasting autonomous responses to negative pictures compared to positive pictures than high PS individuals do. These results are in line with the fMRI results on negative vs. neutral pictures, showing increased activity in the amygdala and right OFC for negative pictures in low PS subjects. This suggests that low PS subjects tend to respond with greater physiological arousal and activation of brain regions involved in arousal for negative emotions. This result can also be interpreted in light of the positive vs. negative picture contrast, which showed decreased activity in the hippocampus. This is compatible with a relative decrease in arousal for the positive pictures in the low PS group. This finding should be confirmed in a larger sample.

### 4.1 Greater emotional vulnerability and avoidance in low persistence subjects

When viewing negative vs. neutral pictures, low PS subjects (compared to high PS) showed higher activity in the amygdala. Abnormal amygdala response to aversive information has been documented across various psychiatric conditions such as mood disorders [48] and anxiety disorders [e.g., 49]. Whereas our low PS subjects did not differ from high PS subjects on depression and anxiety scores, the higher amygdala reactivity to negative pictures could instead indicate greater emotional vulnerability to negative information in low PS subjects. This hypothesis is consistent with Teasdale's differential activation hypothesis (DAH) of depression [50] and recent theories on depressive rumination [51]. DAH theory, which is empirically supported by both cross-sectional and prospective studies, holds that the key factors that determine whether an initial depression becomes more severe or persistent are the degree of activation and the content of negative thinking patterns that become accessible in the depressed state (i.e., cognitive reactivity) [50]. Our results suggest that the propensity to greater emotional reactivity to a negative stimulus could be present even in the absence of clinical symptoms of low mood or depression. Early detection of this propensity could have a major impact, as cognitive reactivity was considered as a potential causal risk factor for depressive relapse or recurrence, and possibly for suicidal relapse or recurrence [50]. Moreover, according to Taesdale‘s hypothesis, mindfulness-based cognitive therapy was found to significantly diminish depressive rumination [52]. A meta-cognitive strategy could therefore build emotional regulation capacities in low PS individuals [53].

Low PS subjects also showed strong hemispheric asymmetry in the OFC response to negative pictures, particularly in the lateral and anterior portions of the OFC, with higher activity in the right OFC and lower activity in the left OFC. This asymmetry suggests activation of an approach/avoidance scheme, whereby left frontal regions contribute to approaching positive stimuli and right frontal regions to avoiding negative stimuli [26]–[28]. Specifically, the lateral anterior OFC was previously related to the evaluation of punishers, which may lead to a change in ongoing behaviour [54], and might be involved in emotional regulation strategies, mainly for negative emotions in the right hemisphere [26]–[28]. It is also known that damage to the OFC may cause significant changes in personality, social conduct, and emotion regulation processes [54]. Moreover, structural OFC abnormalities were found in major depressive disorder (MDD) patients compared to controls [55], and more markedly, in patients with previous suicide attempts [56], suggesting that this region plays a role in the pathophysiology of MDD and suicidal behaviours. Taken together, this pattern of results suggests that low PS individuals show a general increase in brain reactivity in response to negative pictures involving a distributed set of brain areas, including the OFC [23] (with hemispheric asymmetry in favour of the right side [3]) and the amygdala, which is in a position to modulate early sensory processing through top-down influences [57]. Increased activity in a set of areas involved in the processing and regulation of negative emotions suggests that low PS individuals could also be more vulnerable to negative emotions, and potentially to depression.

### 4.2 Decreased activity in the hippocampus in low persistent subjects for positive stimuli

Low (vs. high) PS subjects showed lower activity in the hippocampus when positive (vs. negative) pictures were displayed. Given that the hippocampus is involved in memory functions [58], this result suggests less efficient memory formation for positive emotions in low PS subjects, and that PS could be associated with the degree of persistence of positive or motivation-related emotions in memory. This result is also consistent with a recent study that showed a positive correlation between PS temperament and grey matter concentration in the parahippocampal gyrus [12]. Moreover, differential activation of the hippocampus at encoding acts as a signal for the subsequent consolidation of relevant memories, and is associated with better ability to remember these stimuli [59], [60].

In addition to reduced activation of the hippocampus, low PS (vs. high PS) subjects showed lower activity in the amygdala for positive vs. negative pictures, further supporting our hypothesis that positive pictures may induce lower emotional responses in low PS subjects.

### 4.3 Lower amygdala and dorsal striatum activity in low persistence subjects

When a neutral stimulus was presented, low PS subjects (vs. high PS subjects) showed lower activity in a brain area involved in emotion and reward processing (i.e., the caudate nucleus [61]). This result suggests that in a non-emotional condition, high PS individuals would be more likely to engage in self-rewarding behaviours, even in the absence of externally rewarding stimuli, unlike low PS individuals. This finding is consistent with the highly positive affects found in high PS subjects in a recent study [62], and corroborates the results by Gusnard et al. [11] showing that with increased proportion of neutral pictures and decreased external arousals and external rewards, high PS subjects showed increased activity in the orbital and adjacent mPFC, suggesting the generation and maintenance of internal arousal [11]. The orbital and adjacent mPFC, amygdala, and caudate are part of a mesolimbic reward network [63]. In addition, a recent meta-analysis of 142 neuroimaging studies that examined brain activation in reward tasks in healthy adults showed that the amygdala, caudate, and orbital and adjacent mPFC belong to a core circuit involved in reward-related decision making [64]. Moreover, the amygdala and orbitofrontal cortex have been shown to be involved in reward outcome [64]. Consistent with Gusnard et al.'s study [11], our results suggest a deficit in an internal motivation maintenance network in low PS subjects.

### 4.4 Changes in functional connectivity in high and low PS subjects

In order to refine our investigation of the underlying brain mechanisms in the differential activation of cerebral areas when emotional stimuli are presented, we assessed the functional connectivity of key regions revealed in the main analysis and other regions across the whole brain using psychophysiological interactions (PPI). In high PS subjects, functional coupling was increased between the left OFC area (found in low vs. high PS subjects for the negative> neutral contrast) and the visual cortex. For connectivity with the visual cortex, this result could reflect increased visual cortex activity in response to negative stimuli, but only for high PS subjects, which might serve as a better control over the early visual area in order to manage emotions elicited by negative pictures. Taken together, these results suggest that high PS subjects responding to negative pictures activate a network that decreases the impact of negative emotions on visuo-limbic areas.

We also found increased functional connectivity between the right amygdala (in low vs. high PS for the negative> neutral contrast), visual cortex, and anterior insula. This pattern of results is consistent with other studies suggesting anatomical [57] and functional [65] connections between the amygdala and the visual cortex to mediate enhanced processing of emotionally relevant material [65]. Moreover, increased connectivity between the amygdala and the insular cortex suggests better interoceptive processing of emotional stimuli that may threaten bodily integrity [25]. For positive stimuli, functional connectivity was greater between the amygdala and the OFC in high (vs. low) PS subjects, which may enable more efficient valuation of rewarding stimuli and reinforce motivation, which could explain the greater behavioural perseverance in high PS subjects. This study shows that a network of areas (i.e., the amygdale, insula, and OFC) involved in emotion regulation and interoception is differentially involved as a function of individual PS.

### 4.5 Persistence as a state effect or trait vulnerability marker

Cloninger et al. [66] showed that a high PS score can be considered as a trait. In particular, high PS is positively (but weakly) associated with the occurrence of depressive symptoms [66] and risk for depression [67]. Thus, highly persistent individuals are proposed to be overachieving and to drive themselves far beyond what is necessary. These are attributes that may promote the development of depression [68] and anxiety [69]. In contrast, some studies found that depressed patients have a low PS score, but that patients in remission have comparatively higher PS scores [15]. Moreover, there is evidence that PS correlates inversely with depression scores [70], [71]. This would suggest that PS could also be modulated by mood state. Whereas these studies appear to suggest that everyone is at risk for depression regardless of PS score, studies that address vulnerability to psychopathology generally only refer to the extreme ends of the PS spectrum.

According to the above-cited studies, it is therefore likely that PS interacts with the environment and that low and high PS lead to distinct responses to daily challenges. Moreover, it remains to be clarified whether PS can be generalized to all types of behaviour [20]. In particular, in the long term, high PS individuals could present more severe dysfunctional behaviours that could develop into depression or anxiety, whereas low PS subjects could present decreased coping abilities that could also develop into depression. This suggests that both low and high PS may give rise to depression, but in different ways, which could be depicted on a U-curve with depression on the ordinate axis and persistence on the abscissa axis.

Our study considers PS as a relatively stable personality trait that can modulate brain responses to emotional stimuli. Note also that, in our study, low and high PS subjects did not differ on depression and anxiety scores, making it unlikely that our main results were confounded by mood or anxiety factors.

### 4.6 The partial reinforcement extinction effect (PREE), and low and high PS as both adaptive and maladaptive strategies

Cloninger proposed that PS could be measured by the PREE, because PS subjects would be more resistant to extinction of previously intermittently rewarded behaviours than control subjects who have been continuously reinforced (CR) [13], [18]. The present study found that low PS (vs. high PS) subjects had lower activity in the hippocampus when responding to positive (vs. negative) pictures. This original result is of interest because PREE disruption has been shown to be caused by projections from the hippocampus to the nucleus accumbens in rodents [21]. The disengagement of the hippocampus in low PS extends the results of Gusnard et al., who demonstrated the involvement of the nucleus accumbens [11].

Some methodological considerations and limitations of this study should be noted. Unlike Gusnard et al. [11], we used an event-related fMRI design and not a block design to assess the influence of the emotions associated with the displayed pictures (positive, negative, or neutral) when self-rated by subjects. This method allowed us to assess as closely as possible the subject's emotional response to each displayed picture. We used self-report questionnaires to measure depression, anxiety, and dimensions of temperament. Although the validity and reliability of these instruments have been established, there remain differences between the results of self-report questionnaires and semi-structured interviews. Furthermore, as our findings are relevant for individual variability in healthy volunteers, it would be instructive to adopt a similar approach to examine PS as a trait marker in clinical populations such as depressive patients. Finally, in future studies it would be useful to determine how PS relates to other cognitive measures by adding measures of cognition (e.g., attention, working memory) and other personality dimensions (e.g., neuroticism, attachment).

## Conclusion

Our results reveal that a personality trait, low PS, can increase the amygdala and right OFC response to negative emotions, modulate frontal top-down appraisal processes, and decrease hippocampus activity when pleasant stimuli are presented. This pattern of results could suggest less memory for pleasant events, plausibly leading to impaired generation and maintenance of internal motivation (i.e., low PS). Conversely, our results also show that high PS subjects recruit mechanisms of frontal top-down control over the amygdala when negative stimuli are presented, consistent with efficient regulation of the emotional response to aversive signals. These high PS subjects would also better remember positive information due to greater hippocampus activation when positive pictures are presented. Finally, high PS subjects showed higher activation of the amygdala and dorsal striatum, which may suggest that they are better able maintain internal arousal even in the absence of positive or rewarding external stimulation. These results are promising for the management of individual differences that make subjects differentially vulnerable to affective disorders such as depression, and which could be used as endophenotypic markers of emotional vulnerability.

## Supporting Information

Figure S1 **Protocol.**
(TIF)

Figure S2 **Regions more connected to seed areas [(A) right amygdala, (B) left orbitofrontal cortex] for negative than neutral pictures.** The parameter estimates are calculated for negative (Neg) and neutral (Neu) items. arbitrary units, error bars: SEM). **A.** The strength of connectivity with amygdala is larger in the anterior insula and the fusiform gyrus more for low PS subjects than high PS subjects **B.** The cuneus and the superior lingual gyrus are more connected to OFC for high PS subjects than low PS subjects (Functional results are displayed on the mean structural MR image of the participants normalized to the MNI stereotactic space (display at p, 0.001, uncorrected).
(TIF)

File S1 **Contains supporting tables.** Table S1, Behavioral and physiological results. Table S2, Psychophysiological interaction on seed areas for the contrast negative vs neutral pictures. Table S3, Contrast Negative versus neutral images, all subjects. Table S4, Contrast negative versus positive images all subjects. Table S5, Contrast positive versus neutral images all subjects. Table S6, Contrast positive versus negative images all subjects.
(DOC)
