# Supplementary figures and images for: Ability to Maintain Internal Arousal and Motivation Modulates Brain Responses to Emotions
Source: PLoS One. 2014 Dec 1;9(12):e112999. doi: 10.1371/journal.pone.0112999 (PMC4249829; doi:10.1371/journal.pone.0112999)

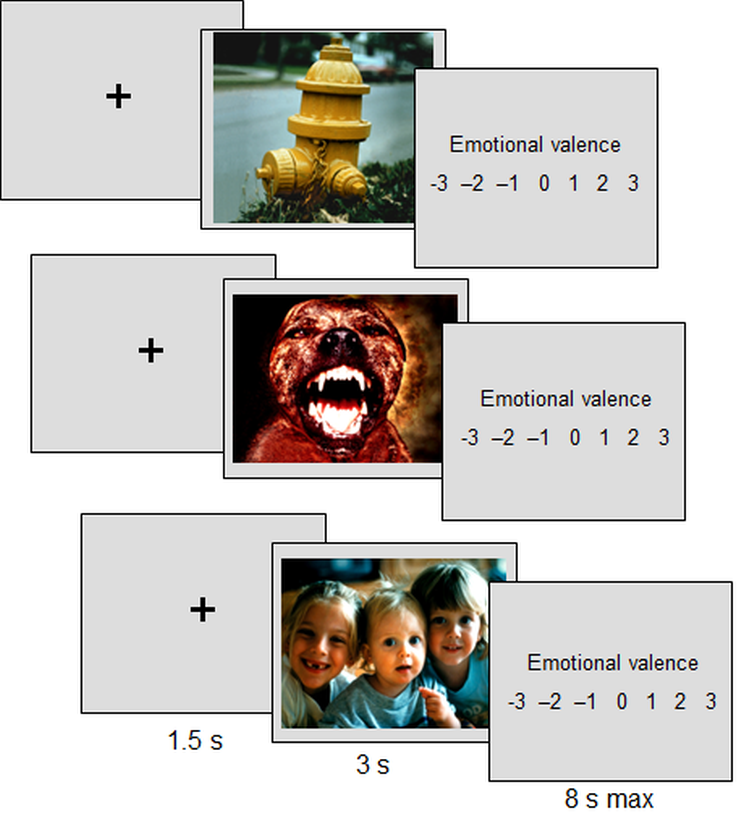

Supplement: Figure S1 — Protocol. (TIF) [file pone.0112999.s001.tif]

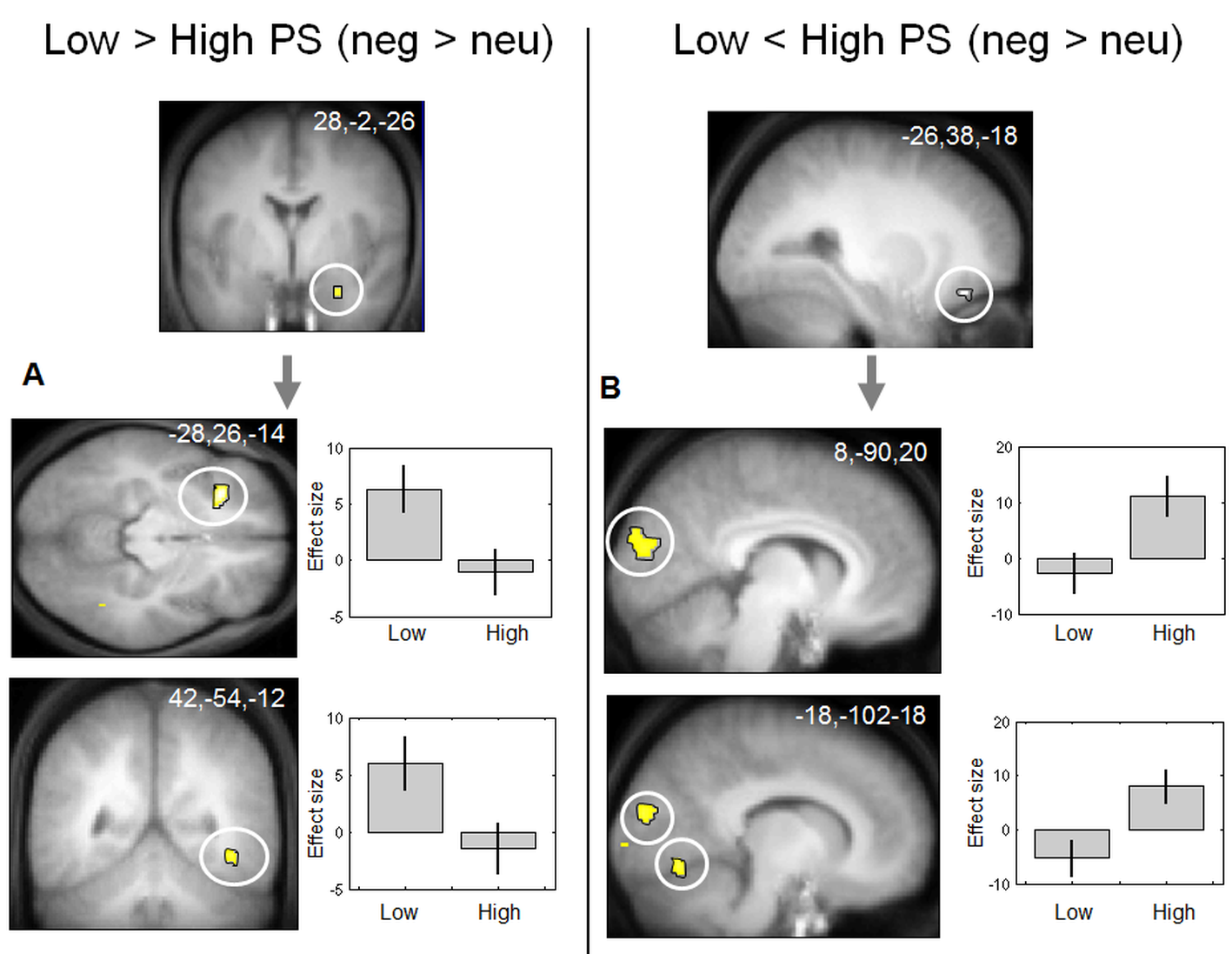

Supplement: Figure S2 — Regions more connected to seed areas [(A) right amygdala, (B) left orbitofrontal cortex] for negative than neutral pictures. The parameter estimates are calculated for negative (Neg) and neutral (Neu) items. arbitrary units, error bars: SEM). A. The strength of connectivity with amygdala is larger in the anterior insula and the fusiform gyrus more for low PS subjects than high PS subjects B. The cuneus and the superior lingual gyrus are more connected to OFC for high PS subjects than low PS subjects (Functional results are displayed on the mean structural MR image of the participants normalized to the MNI stereotactic space (display at p, 0.001, uncorrected). (TIF) [file pone.0112999.s002.tif]
