# Supplementary material for: Ability to Maintain Internal Arousal and Motivation Modulates Brain Responses to Emotions
Source: PLoS One. 2014 Dec 1;9(12):e112999. doi: 10.1371/journal.pone.0112999 (PMC4249829; doi:10.1371/journal.pone.0112999)
Supplement: File S1 — Contains supporting tables. Table S1, Behavioral and physiological results. Table S2, Psychophysiological interaction on seed areas for the contrast negative vs neutral pictures. Table S3, Contrast Negative versus neutral images, all subjects. Table S4, Contrast negative versus positive images all subjects. Table S5, Contrast positive versus neutral images all subjects. Table S6, Contrast positive versus negative images all subjects. (DOC) [file pone.0112999.s003.doc]

**Ability to maintain internal arousal and motivation modulates brain responses to emotions**

Table S1 : **Behavioral and physiological results**

|  | **Negative** | **Neutral** | **Positive** |
| --- | --- | --- | --- |
| **Number of images identified for each emotion (for 120 pictures)** | | | |
| **High PS** | 30.9 ± 5.6 | 58.1 ± 14.2 | 30.9 ± 11.1 |
| **Low PS** | 28.6 ± 10.1 | 64.3 ± 18.3 | 27.4 ± 10.8 |
| **Mean reaction time for each emotion (ms)** | | | |
| **High PS** | 903 ± 404 | 1073 ± 467 | 903 ± 319 |
| **Low PS** | 795 ± 305 | 901 ± 258 | 808 ± 224 |
| **Mean objective emotional arousal measured by pupillary size** | | | |
| **High PS** | 0.47 ± 0.16 | 0.39 ± 0.16 | 0.39 ± 0.17 |
| **Low PS** | 0.43 ± 0.24 | 0.34 ± 0.20 | 0.32 ± 0.23 |

**Table S2 :** Psychophysiological interaction on seed areas for the contrast negative vs neutral pictures

| **Name of the brain regions** | **MNI coordinates**  **(x,y,z:mm)** | **Side** | **Z-score** | **Voxel size** | **P_svc_** | **Coordinates from the literature** | |
| --- | --- | --- | --- | --- | --- | --- | --- |
| **PPI on the amygdala, Low > High PS** | | | | | | |  |
| Anterior insula | -28, 26, -14 | L | 4.33 | 106 | 0.001 | 30,20,-8 | |
| Fusiform gyrus | 42,-54,-12 | R | 3.32 | 11 | 0.029 | 44,-54-20 | |
| **PPI on the orbitofrontal cortex, Low < High PS** | | | | | | |  |
| Superior occipital gyrus | 8,-90,20  -26,-90,6 | R  L | 3.36  3.16 | 19  5 | 0.028  0.048 | 2,-82,20  –28, -84, 0 | |
| Lingual gyrus | 14,-72,-8 | R | 3.47 | 48 | 0.021 | -20, -74, -8 | |
| Cuneus | -18,-102,18 | R | 3.76 | 133 | 0.007 | -17,-95,-12 | |

**Table S3 :** Contrast Negative versus neutral images, all subjects

| **Name of the brain regions** | **MNI coordinates**  **(x,y,z:mm)** | **Side** | **Z-score** | **Voxel size** | **P_svc_** |
| --- | --- | --- | --- | --- | --- |
| Middle temporal gyrus | 52, -60, 2  -48, -60, 8 | R  L | 6.38  6.12 | 3160  5818 | <0.001  <0.001 |
| Middle occipital gyrus | 52,-78,2  -50,-78,0 | R  L | 5.44  6.07 | 5818  5818 | 0.003  <0.001 |
| Fusiform gyrus | 44,-48,-22  -42,-40,-22 | R  L | 5.33  4.72 | 3160  5818 | 0.004  <0.001 |
| Amygdala | 26,-8,-16 | R | 5.75 | 3593 | 0.001 |
| Thalamus | 4,-30,-10 | R | 5.44 | 847 | 0.003 |
| Orbitofrontal cortex | 0,56,-16 | R | 5.30 | 399 | 0.005 |
| Medial prefrontal cortex | -6,46,32  8,58,26 | L  R | 5.00  5.47 | 1818  1818 | 0.019  0.002 |
| Precentral gyrus | 40,-22,56 | R | 6.94 | 4106 | <0.001 |
| Cingulate sulcus | 8,0,42  2,-50,22 | R  R | 5.20  5.13 | 880  758 | 0.008  0.011 |
| Putamen | 30,-10,-10 | R | 5.84 | 3593 | <0.001 |
| Inferior frontal gyrus | 52,32,2 | R | 4.45 | 118 | 0.001 |

Table S4 : **Contrast negative versus positive images all subjects**

| **Name of the brain regions** | **MNI coordinates**  **(x,y,z:mm)** | **Side** | **Z-score** | **Voxel size** | **P_svc_** |
| --- | --- | --- | --- | --- | --- |
| Middle occipital gyrus | -52,-76,0  42,-78,0 | R  L | 6.18  3.55 | 1814  485 | <0.001  0.016 |
| Fusiform gyrus | -40,-42,-20 | L | 4.43 | 1814 | 0.001 |
| Medial prefrontal cortex | 8,64,30  -6,44,30 | R  L | 3.10  4.55 | 190  190 | 0.049  0.001 |
| Precentral gyrus | 40,-22,58 | R | 7.43 | 4896 | <0.001 |
| Inferior frontal gyrus | -40,24,-20 | L | 4.04 | 34 | 0.012 |

Table S5 : **Contrast positive versus neutral images all subjects**

| **Name of the brain regions** | **MNI coordinates**  **(x,y,z:mm)** | **Side** | **Z-score** | **Voxel size** | **P value** |
| --- | --- | --- | --- | --- | --- |
| Middle temporal gyrus | 66,-36,20 | R | 5.01 | 2395 | 0.016 |
| Middle occipital cortex | 54,-74,8  -46,-62,12 | R  L | 4.79  4.56 | 2395  38115 | 0.022  0.033 |
| Cuneus | 4,-90,28 | R | 5.32 | 38115 | 0.001 |
| Amygdala | -24,-2,-18 | L | 5.76 | 38115 | <0.001 |
| Thalamus | -6,-28,-14 | L | 4.53 | 38115 | 0.020 |
| Insula | -42,2,0  38,8,4 | L  R | 5.21  4.38 | 38115  38115 | 0.001  0.035 |
| Medial prefrontal cortex | -10,56,6 | L | 6.02 | 38115 | <0.001 |
| Precentral gyrus | -36,-24,72 | L | 6.98 | 38115 | <0.001 |
| Anterior cingulate sulcus | -12,40,-6 | L | 6.75 | 38115 | <0.001 |
| Middle cingulate gyrus | -2,-4,42 | L | 5.59 | 38115 | 0.001 |
| Posterior cingulate gyrus | -4,-50,18 | L | 5.13 | 38115 | 0.010 |
| Putamen | -8,6,-8  8,-4,-6 | L  R | 5.85  5.08 | 38115  38115 | <0.001  0.012 |
| Inferior frontal gyrus | -52,2,-2  64,8,2 | L  R | 5.31  4.42 | 38115  38115 | 0.004  0.030 |

Table S6 : **Contrast positive versus negative images all subjects**

| **Name of the brain regions** | **MNI coordinates**  **(x,y,z:mm)** | **Side** | **Z-score** | **Voxel size** | **P_svc_** |
| --- | --- | --- | --- | --- | --- |
| Inferior occipital cortex | 10,-84,-14 | R | 5.63 | 36719 | 0.001 |
| Cuneus | -2,-100,18 | L | 5.74 | 36719 | 0.001 |
| Thalamus | -16,-22,6 | L | 5.60 | 36719 | 0.001 |
| Insula | -42,-16,16 | L | 7.65 | 36719 | <0.001 |
| Precentral gyrus | -38,-26,70  -36,-22,56 | L  L | Inf  7.80 | 36719  36719 | <0.001  <0.001 |
| Anterior cingulate sulcus | -18,32,-6 | L | 5.71 | 36719 | 0.001 |
| Middle cingulate gyrus | -6,-8,54 | L | 7.29 | 36719 | <0.001 |
| Parietal cortex | -14,-68,58 | L | 5.11 | 36719 | 0.011 |
| Putamen | -28,-10,-4 | L | 6.13 | 36719 | <0.001 |
| Lateral frontal gyrus | -36,50,6 | L | 5.02 | 36719 | 0.016 |
| Cerebellum | 18,-52,-24 | R | Inf | 36719 | <0.001 |

**References**

1. Lewis PA, Critchley HD, Rotshtein P, Dolan RJ (2007) Neural correlates of processing valence and arousal in affective words. Cereb Cortex 17: 742-748.

2. Vuilleumier P, Armony JL, Driver J, Dolan RJ (2001) Effects of attention and emotion on face processing in the human brain: an event-related fMRI study. Neuron 30: 829-841.

3. Sterpenich V, Albouy G, Darsaud A, Schmidt C, Vandewalle G, et al. (2009) Sleep promotes the neural reorganization of remote emotional memory. J Neurosci 29: 5143-5152.

4. Smith AP, Henson RN, Dolan RJ, Rugg MD (2004) fMRI correlates of the episodic retrieval of emotional contexts. Neuroimage 22: 868-878.

5. Simpson JR, Ongur D, Akbudak E, Conturo TE, Ollinger JM, et al. (2000) The emotional modulation of cognitive processing: an fMRI study. J Cogn Neurosci 12 Suppl 2: 157-170.
